# Differential outcomes of TLR2 engagement in inflammation‐induced preterm birth

**DOI:** 10.1002/JLB.3MA0717-274RR

**Published:** 2017-12-28

**Authors:** Monica Cappelletti, Matthew J. Lawson, Calvin C. Chan, Adrienne N. Wilburn, Senad Divanovic

**Affiliations:** ^1^ Division of Immunobiology Cincinnati Children's Hospital Medical Center, and the University of Cincinnati College of Medicine Cincinnati Ohio USA; ^2^ Molecular, Cellular and Biochemical Pharmacology Graduate Program University of Cincinnati College of Medicine Cincinnati Ohio USA; ^3^ Medical Scientist Training Program, University of Cincinnati College of Medicine, Cincinnati, Ohio, USA; ^4^ Immunology Graduate Program Cincinnati Children's Hospital Medical Center and the University of Cincinnati College of Medicine Cincinnati Ohio USA

**Keywords:** Inflammation, TLR2, Preterm Birth, IL‐6, IFN‐b

## Abstract

Preterm birth (PTB) is the leading cause of neonatal mortality worldwide. Infection and inflammation are considered main causes of PTB. Among multiple pathogens, Gram‐positive bacteria are commonly linked with induction of PTB. Although activation of innate immune responses, via TLR2 engagement, by Gram‐positive bacteria is a likely cause, whether induction of PTB depends on the potency of specific microbial components to induce Toll‐like receptor (TLR)2‐driven inflammation has not been elucidated. Here, we show that TLR2 activation by synthetic lipopeptides, Pam2Cys, and Pam3Cys specifically, variably influenced inflammation and subsequent induction of PTB. Pam2Cys challenge, compared to Pam3Cys, induced PTB and promoted significantly higher expression of inflammatory cytokines, specifically IL‐6 and IFN‐β, both *in vivo* and *in vitro*. Notably, antibody‐mediated neutralization of IL‐6 or genetic deletion of type I IFN receptor (IFNAR) was sufficient to protect from Pam2Cys‐driven PTB and to temper excessive proinflammatory cytokine production. Conversely, IFN‐β or IL‐6 was not sufficient to promote induction of PTB by Pam3Cys. In summary, our data implies a divergent function of TLR2‐activating lipopeptides in the magnitude and type of ligand‐driven inflammatory vigor in induction of PTB.

**Abbreviations**
GBS: Group B streptococci
EPM: elicited peritoneal macrophage
PAMP: pathogen‐associated molecular pattern
PTB: preterm birth; i.p., intraperitoneal

## 1 INTRODUCTION

Preterm birth (PTB) is a leading cause of infant morbidity and mortality worldwide.^1^, ^2^ Although the etiology of PTB is multifactorial and remains largely enigmatic, it is well accepted that maternal inflammation, driven by infectious or noninfectious triggers, can play a critical role in preterm labor.^3^ In fact, inflammation and immune activation are paramount for uterine activation and the onset of labor.^2^ Pregnant women are more susceptible to infection by multiple pathogens, which increases the risk of severe illness and adverse pregnancy outcome.^2^ Infections by Gram‐positive bacteria have the most deleterious outcomes during pregnancy.^3^, ^4^ *Listeria monocytogenes*, a Gram‐positive bacteria and common contaminant of a variety of raw foods, has tropism for the placenta and is known to cause PTB in humans and mice.^3^, ^5^ Moreover, Group B streptococci (GBS), the β‐hemolytic, Gram‐positive constituent of the normal vaginal microflora, is frequently associated with PTB.^6^ Of note, Listeria species and GBS are pathogens that drive robust induction of proinflammatory cytokines including, IL‐6, TNF, IFN‐β, and IFN‐γ.^6^, ^7^, ^8^, ^9^, ^10^, ^11^ Ureaplasma species (*Ureaplasma parvum* and *Ureaplasma urealyticum*) are Gram‐positive bacteria commonly isolated from the female reproductive tract and in the amniotic fluid of pregnant women.^12^ However, the contribution of Ureaplasma species to PTB is somewhat controversial.^12^, ^13^, ^14^, ^15^ Size variation of the surface‐exposed lipopeptides on Ureaplasma species has been proposed to affect its ability to interact with innate immune receptors and impact consequent potency of immune response.^15^ Hence, underlying causes of the propensity for certain Gram‐positive bacterial species to trigger PTB are not well understood.

TLRs are a family of evolutionarily conserved innate immune receptors essential for recognition of microbial products.^16^ Activation of TLR signaling by conserved microbial molecular structures leads to cytokine production—via activation of NF‐kB, MAPK (JNK, p38, ERK), and IRF signaling pathways, leading to proinflammatory (e.g., TNF‐α, IL‐8, IL‐6, IFN‐γ, and IFN‐β), anti‐inflammatory (e.g., IL‐10), and immunoregulatory (e.g., IL‐12) cytokine production.^17^, ^18^ TLRs are robustly expressed at the maternal/fetal interface by the pregnant uterus, placenta, amniotic membranes, and trophoblast.^19^, ^20^, ^21^, ^22^, ^23^ In mice, challenge with diverse TLR ligands—via intraperitoneal, intrauterine, or intraamniotic routes—increases proinflammatory cytokine release in the uterus^5^ and fetal membranes,^24^, ^25^ recruits immune cells into the cervix,^26^, ^27^ and induces PTB.^27^, ^28^, ^29^, ^30^, ^31^ Notably, TLR2 is involved in the recognition of lipoteichoic acid and lipopeptides from Gram‐positive bacteria. Specifically, the ability of TLR2 to heterodimerize with either TLR6 or TLR1 results in the recognition of diacylated and triacylated lipopeptides, respectively, hinting at a potential mechanism to discriminate among various microbial ligands and to elicit varied downstream inflammatory responses. However, the contribution of specific TLR2‐activating lipopeptide species in the context of PTB has not been examined.

In this study, we hypothesized that induction of PTB depends on the potency of specific lipopeptides to induce TLR2‐driven inflammation. Our data demonstrate that recognition of diacylated lipopeptide (e.g., Pam2Cys) induced PTB and greater proinflammatory cytokine production compared to triacylated lipopeptide (e.g., Pam3Cys). Our data also suggest that sufficiency of inflammatory mediators to induce PTB following Pam2Cys or Pam3Cys challenge is varied. In sum, these data argue that TLR2‐driven induction of PTB might depend on the magnitude and milieu of ligand‐driven inflammatory vigor and such differences might shed light on why only certain Gram‐positive bacterial species are associated with induction of PTB.

## 2 MATERIALS AND METHODS

### 2.1 Reagents

All cell culture reagents were endotoxin free to the limits of detection of the Limulus amebocyte lysate assay (Lonza, Visp, Switzerland) at the concentrations employed. All TLR ligands (Pam3Cys and Pam2Cys) used were of ultrapure grade (Invivogen, San Diego, CA).

### 2.2 Mice

Animals were housed in a specific pathogen‐free animal facility at CCHMC and handled in high‐efficiency particulate‐filtered laminar flow hoods with free access to food and water. All studies were performed in accordance with the procedures outlined in the Guide for the Care and Use of Laboratory Animals and approved by the CCHMC Institutional Animal Care and Use Committee. Female mice (WT, IFNAR^−/−^), on a C57BL/6J background, were mated with fertile male mice of the same strain. The presence of a vaginal plug was considered at day 1 of pregnancy. Parturition events were monitored on days 17–21 of gestation and defined as complete delivery of pups.

### 2.3 Preterm birth

On day 16 of gestation, gravid female mice were mock‐challenged (saline) i.p. or challenged with Pam3Cys and Pam2Cys at indicated concentrations and treated with IL‐6‐neutralizing antibody (500 μg/mouse; clone MP5‐20F3; BioXCell, West Lebanon, NH) or recombinant mouse IL‐6 (eBioscience, San Diego, CA) as indicated. Exogenous administration of 10^4^ U/mouse IFN‐β (PBL Biomedical Laboratories, Piscataway, NJ) was performed 4 h prior to Pam3Cys and Pam2Cys challenge and IL‐6‐neutralizing antibody administration. PTB was defined as parturition within 24 h after TLR ligand challenge (all pups deceased). Term birth was defined as parturition between days 19 and 21 of gestation (all pups alive).

### 2.4 Cytokine quantification

*In vivo* systemic IL‐6, TNF, and IFN‐γ levels were quantified using *in vivo* cytokine capture assay.^5^, ^32^, ^33^ Briefly, biotinylated capture antibodies (eBioscience) were injected i.p. 3 h prior to Pam3Cys and Pam2Cys administration and serum cytokine levels were determined 4 h later.

*In vitro* murine thioglycollate elicited peritoneal macrophages (EPMs) were generated using standard protocol.^5^, ^34^ EPMs (1 × 10^6^ cells/well) were cultured with or without IFN‐β (250 U/ml) for 4 h, subsequently mock‐stimulated or stimulated with Pam3Cys (1.5 μg/ml) or Pam2Cys (1.5 μg/ml) for 4 h to determine mRNA expression, or with Pam3Cys (100 ng/ml) or Pam2Cys (100 ng/ml) for 18 h to determine cytokine production by ELISA (BD Biosciences, San Diego, CA).

### 2.5 Type I IFN activity quantification

Type I IFN activity in mouse serum samples or cell culture supernatants was measured with reference to a recombinant mouse IFN‐β using an L‐929 cell line transfected with an IFN‐sensitive luciferase construct as previously described.^5^, ^35^ Luciferase activity was measured on a luminometer (SpectraMax L; Molecular Devices, Sunnyvale, CA).

### 2.6 Gene expression

For quantification of mRNA expression in murine samples, cells/tissues were homogenized in TRIzol (Invitrogen, San Diego, CA), RNA was extracted, and cDNA was generated and quantified as previously described^32^ using Light Cycler 480 II (Roche, San Francisco, CA). The following murine primers were utilized:

IL‐6: TGGTACTCCAGAAGACCAGAGG and AACGATGATGCACTTGCAGA,
TNF: CCAGACCCTCACACTCAGATCA and CACTTGGTGGTTTGCTACGAC.
IFNB1: TCCAGCTCCAAGAAAGGACG and TTGAAGTCCGCCCTGTAGGT,
MX‐1: CTCAGGGTGTCGATGAGGTC and TCTGAGGAGAGCCAGACGAT,
IRF9: CACTCGGCCACCATAG and AAGCCATCTCTTTCCAAGTCTTT,
ISG15: GTCACGGACACCAGGAAATC and AAGCAGCCAGCCGCAGACTG.

### 2.7 Statistical analysis

Data were analyzed by unpaired Student *t* test in Prism 5a (GraphPad Software Inc., La Jolla, CA) as appropriate. A *P* value less than 0.05 was considered significant. *In vivo* serum cytokine values were normalized to Pam2Cys challenge as appropriate and expressed as the percent (%) change. All values are represented as means ± se or as percent of term or PTB induction.

## 3 RESULTS AND DISCUSSION

### 3.1 Activation of TLR2 by Pam2Cys but not Pam3Cys induces PTB

**Figure 1:**
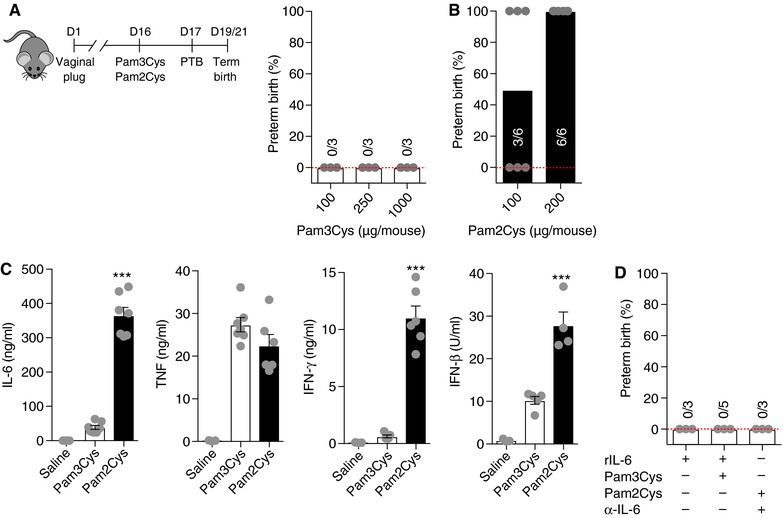
**Activation of TLR2 by Pam2Cys but not Pam3Cys induces preterm birth**. Gravid WT mice challenged with (A) Pam3Cys or (B) Pam2Cys at the indicated doses on day 16 of gestation and the incidence of PTB was quantified. (C) WT mice (*n* = 4–7/condition) were challenged with saline, Pam3Cys (100 μg/mouse) or Pam2Cys (100 μg/mouse) for 4 h, and serum IL‐6, TNF, IFN‐γ, and IFN‐β levels were quantified by *in vivo* cytokine capture assay (IVCCA) and type I IFN activity assay, respectively. (D) Gravid WT mice were treated with IL‐6 neutralizing Ab (500 μg/mouse) or recombinant mouse IL‐6 (10 μg/mouse) followed by above‐described challenge (Figure 1B) and the incidence of PTB was quantified. (A, B and D) Data represent percent of induction of PTB. Student's *t* test, ***P < 0.001

Gram‐positive bacteria (e.g., Listeria, GBS, and Ureaplasma species) are pathogens commonly associated with PTB.^5^, ^6^, ^12^, ^13^, ^14^, ^15^ However, competency of certain bacteria to trigger PTB remains unclear. Although all experimental models of PTB have limitations compared to human pregnancy and parturition, mice represent the most common animal model of PTB.^2^ Specifically, in such experimental models, the doses of inflammatory ligands employed are high. The intent being to enable 100% parturition incidence in fully backcrossed mice.^5^, ^36^, ^37^, ^38^, ^39^, ^40^ Notably, that allows investigators to uncover differences between animal genotypes and/or various ligands in comparison to established baseline reads in wild‐type (WT) mice. Here, we utilized a well‐established, tractable, model of PTB induction in mice^5^ for mechanistic insight into how early parturition is triggered in the context of systemic TLR2 challenge (Figure 1A and B). The pathways regulating immune responses are highly conserved between mice and humans—indicating that mice are a useful experimental tool for the interrogation of the role of infection/inflammation in induction of PTB.^21^, ^41^ Further, in mice following i.p. TLR ligand injection, parturition‐associated molecules are upregulated in the entire gestational environment and affect all fetuses.^5^ Hence, the parturition process once initiated results in the delivery of all pups.

To overcome the complexity of a live bacterial infection (e.g., replication rate, tropism for various cell subsets, activation of multiple innate immune receptors), we used purified synthetic lipopeptides known to activate TLR2 signaling—pathogen‐associated molecular patterns (PAMPs) that molecularly mimic the acylated amino terminus of bacterial lipopeptides.^42^ Pregnant mice at day 16 of gestation were protected from PTB even at very high doses of triacylated lipopeptide (Pam3Cys; 1 mg/mouse) challenge (Figure 1A). Conversely, challenge with diacylated lipopeptide (Pam2Cys; 200 μg/mouse) induced 100% PTB at the commonly used doses^43^, ^44^, ^45^ (Figure 1B). In this experimental setting utilizing Pam2Cys challenge, all delivered pups died (either in utero or upon delivery) while the mothers survived the challenge without presenting apparent adverse outcomes that could hinder their health status. In mice, lung maturation is not fully completed at day 16 of gestation.^46^ As our model invokes initiation of inflammatory insult at day 16 of gestation, the lack of lung maturation/function in prematurely born pups may represent the likely locus responsible for fetal death upon premature parturition.^36^

As inflammation is directly linked with TLR‐driven induction of PTB, the capacity of Pam2Cys and Pam3Cys to induce inflammatory responses was examined next. Induction of PTB by Pam2Cys correlated with significantly increased IL‐6, IFN‐γ, and IFN‐β, but similar TNF production compared to Pam3Cys challenge—findings when performed with equivalent doses of these TLR2 ligands (Figure 1C). The central role of proinflammatory cytokines, and IL‐6 in particular, is supported by the correlation between IL‐6 levels and PTB in humans^47^ and in mouse models of TLR‐driven inflammation.^5^, ^38^ Pharmacological inhibition of IL‐6 (antibody mediated) was sufficient to protect from Pam2Cys‐driven PTB, while a single dose of rIL‐6 alone or rIL‐6 in combination with Pam3Cys was not sufficient to trigger induction of PTB (Figure 1D)—something supported by a previously published report.^48^ Such findings suggest that Pam2Cys and Pam3Cys signaling activate divergent immune mediators that may play a role in induction of PTB. Further, these data also suggest that IL‐6 is not the only proinflammatory cytokine upregulated in the context of PTB and that other immune mediators likely play a role in induction of PTB. Specifically, of interest to our findings, published reports suggest a role for IFN‐γ in the regulation of mediators involved in the onset of labor.^49^ Importantly, such findings warrant that future studies should expand the knowledge of IFN‐γ in inflammation‐driven PTB.

Overall, our data suggest that Pam2Cys mediates a more potent TLR2‐driven inflammatory response than Pam3Cys and that IL‐6 induction by Pam2Cys, but not Pam3Cys, plays an important role in PTB. Enhanced solubility of Pam2Cys in saline—something that is dependent not only on the lipid content but also on the position of attachment of the lipids^50^ and a more potent stimulation of splenocytes and macrophages compared to Pam3Cys^51^, ^52^ might be central to the observed effects. Environmental conditions including acidic pH, a postlogarithmic bacterial growth phase, high temperatures, and high salt concentrations are all known to support the accumulation of the diacyl lipopeptides. Hence, such conditions may possibly impact the balance between di‐ versus triacylated lipopeptides expression by certain bacteria and influence the type and vigor of immune responses.^53^ Whether these conditions are present in the intrauterine environment and specifically in the placenta and amniotic fluid during pathological pregnancies, however, needs to be further elucidated. It is plausible that the pregnant uterus might represent a favorable environment for the replication of certain pathogens and might regulate the balance of lipopeptides expression of PTB‐related bacteria, thus directly impacting the type of inflammatory responses by both immune and nonimmune cells.^54^ Of note, *L. monocytogenes* and *Mycoplasma fermentans*, pathogens correlated with PTB in humans, exclusively contains/expresses diacylated lipoproteins, structurally similar to Pam2Cys, in its cell wall.^55^, ^56^ Similarly, *U. parvum*, associated with PTB in humans, similarly express diacylated lipopeptide and are essential for the initiation of inflammation.^57^ Further, the differential ability of lipopeptides to induce inflammation might rely on specific amino acids that affect their potency in inducing TLR2‐dependent responses and on the ability of TLR2 to heterodimerize with either TLR6 or TLR1.^58^ However, the contribution of either TLR6 or TLR1 heterodimerization to TLR2 in inflammation‐driven PTB has not been examined. Hence, these gaps in knowledge, coupled with our findings invoking divergent effects of Pam2Cys and Pam3Cys in induction of inflammatory vigor and PTB, highlight the relevance of future studies focused on the role of TLR1 and TLR6 signaling in such biological outcomes. Overall, such factors might posit the specificity of certain Gram‐positive bacterial species to augment immune responses and trigger PTB.

### 3.2 Pam2Cys drives expression of type I IFN signature genes

Type I IFNs, rapidly induced in various cell types upon viral and bacterial infections, represent key signaling cytokines central to activation of both innate and adaptive immune responses. All type I IFNs bind a common receptor at the surface of cells, which is known as the type I IFN receptor. The type I IFN receptor is composed of 2 subunits, IFNAR1 and IFNAR2, which are associated with TYK2 and JAK1, respectively.^59^ The outcomes of Gram‐positive bacterial ligand dependent activation of TLR2 signaling are commonly focused on proinflammatory cytokine production. However, recent studies have demonstrated that, in addition to NF‐kB‐dependent proinflammatory cytokines,^60^ intracellular TLR2 activation by viral and bacterial components (e.g., UV irradiated and live virus, Pam2Cys, Pam3Cys, and MALP‐2, respectively) also leads to the production of IFN‐β‐ and type I IFN‐dependent responses.^45^, ^60^, ^61^ Of note, the type I IFN axis plays a critical role in TLR‐driven PTB and exacerbation of systemic and reproductive site inflammation.^5^ Hence, we examined the robustness of TLR2‐activating lipopeptides to promote IFN‐β production and expression of type I IFN signature genes.

**Figure 2:**
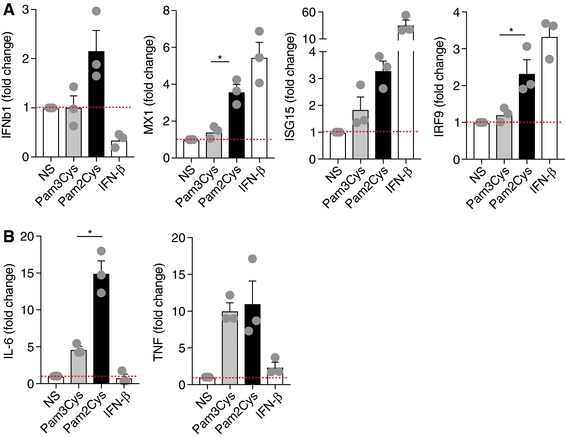
**Pam2Cys challenge promotes type I IFN‐associated gene expression**. WT murine peritoneal macrophages were treated with Pam2Cys (1.5 μg/ml), Pam3Cys (1.5 μg/ml), and IFN‐β (250 U/ml) for 4 h and expression levels of (A) type I IFN signature genes (IFN‐β1, Mx1, Isg15, and Irf9) and (B) proinflammatory cytokines (IL‐6 and TNF) were quantified by PCR. Data are representative of 3 independent experiments. Data are normalized to not stimulated (NS) condition

*In vivo* stimulation by Pam2Cys led to increased IFN‐β production compared to Pam3Cys (Figure 1C). Similarly, *in vitro* stimulation of peritoneal macrophages by Pam2Cys triggered a more robust expression of IFNβ1 and type I IFN signature genes (e.g., Mx1, Isg15, and IRF9) (Figure 2A). Further, this increase was concomitant with enhanced IL‐6 and similar TNF mRNA levels compared to Pam3Cys (Figure 2B). Although the type I IFN family in mice is composed of IFN‐α (with 14 known subtypes) and IFN‐β, TLR2‐driven type I IFN‐dependent responses are believed to be largely dependent on the activity of IFN‐β.^60^ Notably, in agreement with the published report, IFN‐β‐deficient macrophages exhibit reduced IL‐6 expression following Pam2Cys and Pam3Cys stimulation in vitro (data not shown). Moreover, we and others have shown that dysregulation of IFN‐β is a major determinant for PTB in context of a polymicrobial infection.^5^, ^62^ However, the possible contribution of other type I IFNs (e.g., IFN‐α isotypes) has not been formally examined.

Although TLR2 activation was thought to specifically induce NF‐kB‐driven cytokine production, recently the contribution of endolysosomal TLR2 activation has been shown to promote type I IFN production.^60^ However, the contribution of TLR2 homodimerization or dimerization of TLR2 with either TLR1 or TLR6 for induction of type I IFN is not known.^63^ Hence, an improved mechanistic understanding of TLR2/1 and TLR2/6 heterodimeric signaling and downstream pathways in induction of PTB would be of high interest and importance to the research community. Lastly, the activating as well as regulatory mechanisms downstream of TLR2‐dependent induction of type I IFNs need to be elucidated. Thus, TLR2 signaling potentially discriminates a variety of pathogens and regulates the nature of responses against both extracellular and intracellular microbes.

### 3.3 IFNAR signaling contributes to Pam2Cys‐mediated induction of PTB

**Figure 3:**
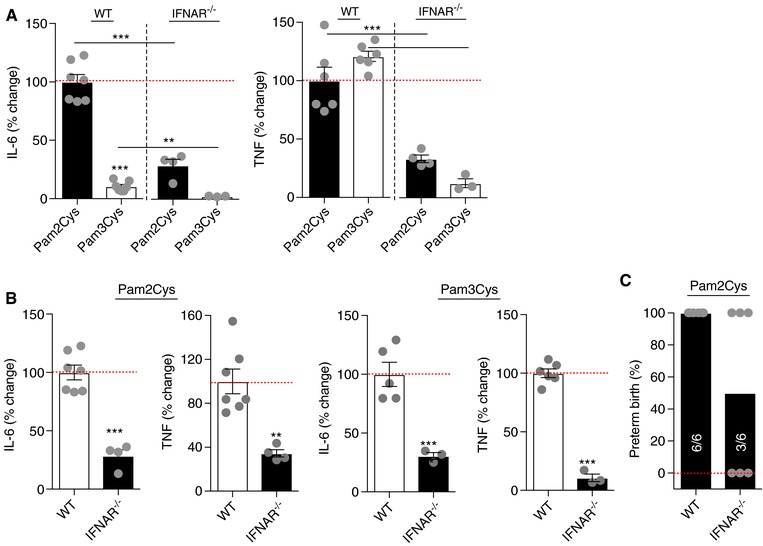
**IFNAR signaling is required for Pam2Cys‐mediated proinflammatory cytokine production and induction of preterm birth**. (A and B) WT and IFNAR^−/−^ mice (*n* = 3–7/condition) were challenged with Pam3Cys (100 μg/mouse) or Pam2Cys (100 μg/mouse) for 4 h and serum IL‐6 and TNF levels were quantified by *in vivo* cytokine capture assay (IVCCA). (C) Gravid WT and IFNAR^−/−^ mice were challenged with Pam2Cys (200 μg/mouse) on day 16 of gestation and the incidence of PTB was quantified. *In vivo* serum cytokine values were normalized to Pam2Cys challenge as appropriate and expressed as the percent (%) change. Student's *t* test, **P < 0.01, ***P < 0.001

Given the robust induction of type I IFN production and signature after Pam2Cys‐driven TLR2 activation, the necessity of the common IFN α/β receptor (IFNAR) in proinflammatory cytokine production and PTB was further investigated.^64^ IFNAR is a heteromeric cell surface receptor with 2 subunits, IFNAR1 and IFNAR2. Of note, IFN‐β specifically interacts with IFNAR1 in an IFNAR2‐independent manner.^65^ Compared to WT‐treated mice, genetic deletion of IFNAR1 tempered Pam2Cys‐dependent induction of IL‐6 and TNF (Figure 3A and B) and resulted in a 50% protection from Pam2Cys‐driven PTB (Figure 3C). The lack of IFNAR1 signaling similarly decreased Pam3Cys‐driven IL‐6 and TNF production. Further Pam2Cys‐driven cytokine production was significantly higher compared to Pam3Cys in both WT and IFNAR^−/−^ mice (Figure 3A and B). These data suggest that both synthetic lipopeptides activate the type I IFN/IFNAR axis and that such activation regulates proinflammatory cytokine production in this context. However, although significant, the protection from Pam2Cys‐driven PTB in IFNAR‐deficient mice was not complete (Figure 3C), indicating that additional signaling pathways activated following Pam2Cys challenge are similarly involved. Hence, how IFNAR signaling (e.g., IFNAR1 and IFNAR2) contributes to protection from Pam2Cys‐driven PTB warrants further investigation.

In fact, IFNAR modulation of chemokine production (e.g., CCL2 and CCL4), proinflammatory cytokines (IL‐1β and IL‐18), inflammasome activation, macrophage infiltration and activation, and uterine tissue inflammation (e.g. Cox‐2 and prostaglandin E_2_) may similarly play a role.^5^, ^66^, ^67^, ^68^, ^69^ The contribution of IFN‐α subtypes and IFN‐β, something that is pathogen specific as well as anatomical locus/tissue/cell type specific,^59^ also remains under defined. However, IFN‐β might have a predominant role as it is an important determinant of pathogen‐related virulence and is robustly induced by Gram‐positive bacteria associated with PTB (e.g. Listeria species, GBS).^7^, ^60^

### 3.4 IFN‐β‐driven enhancement of Pam3Cys‐associated inflammation is not sufficient for induction of PTB

**Figure 4:**
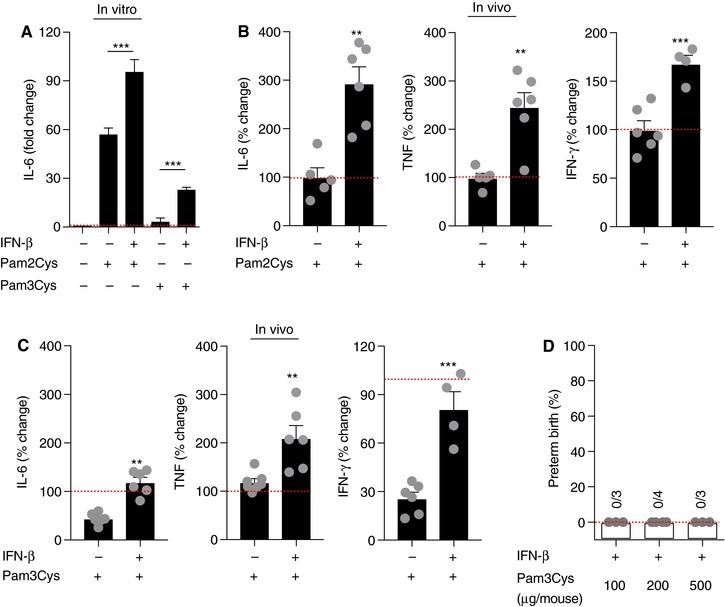
**IFN‐β‐driven enhancement of Pam3Cys‐associated inflammation is not sufficient for induction of preterm birth**. (A) WT murine peritoneal macrophages (*n* = 3) were treated with recombinant mouse IFN‐β (250 U/ml; 4 h) prior to being challenged with Pam2Cys (100 ng/ml) and Pam3Cys (100 ng/ml) for 18 h and IL‐6 levels were quantified by ELISA. (B and C) WT mice (*n* = 3–6/condition) were challenged with recombinant mouse IFN‐β (10^4^ U/mouse for 4 h) prior to being challenged with Pam3Cys or Pam2Cys (25 μg/mouse for 4 h) and serum IL‐6 and TNF levels were quantified by *in vivo* cytokine capture assay (IVCCA). (D) Gravid WT mice were challenged with recombinant mouse IFN‐β (10^4^ U/mouse for 4 h) prior to being challenged with Pam3Cys at the indicated doses on day 16 of gestation and the incidence of PTB was quantified. Cytokine values were normalized to (A) not stimulated control and (B) Pam2Cys or (C) Pam3Cys challenge as appropriate and expressed as the percent (%) change. Student's *t* test, **P < 0.01, ***P < 0.001

A combined recognition of viral and bacterial molecular patterns by TLR, coined a “double hit hypothesis”, has been proposed as a central regulator of increased susceptibility to PTB.^40^, ^70^, ^71^ We have previously demonstrated that type I IFNs regulates TLR4‐driven inflammatory vigor, and specifically primes for exacerbated IL‐6 and TNF production.^5^ As Gram‐positive bacteria‐derived PAMPs are associated with PTB and robust activation of the type I IFN axis, we asked whether type I IFN priming acts as a universal sensitization mechanism to secondary challenge with all TLRs (including those that alone do not induce PTB). Of note, *in vitro* IFN‐β administration was sufficient, although at different levels, to prime for a secondary challenge with Pam2Cys and Pam3Cys (Figure 4A). This was recapitulated *in vivo* as IFN‐β priming exacerbated IL‐6, TNF, and IFN‐γ production by both Pam2Cys and Pam3Cys. However, IFN‐β priming induced significantly higher levels of proinflammatory cytokines upon secondary challenge with Pam2Cys (Figure 4B and C).

The relevance and sufficiency of such effects on PTB was examined next. IFN‐β sensitization was not sufficient to augment Pam3Cys‐driven inflammatory vigor necessary for induction of PTB, even at very high concentration of ligand (Figure 4D). These data suggest that Pam2Cys and Pam3Cys have distinct functional consequences upon the activation of different heterodimers. Whether IFN‐β sensitization is sufficient to lower the necessary threshold for Pam2Cys for induction of PTB, as observed for LPS,^5^ still needs to be defined. Differential contribution of the MyD88‐ and TRIF‐dependent pathways in TLR2‐mediated signal transduction and subsequent induction of gene expression may be responsible for optimal host responses toward certain infections.^72^ Furthermore, multiple factors are known to affect the role of type I IFNs in controlling susceptibility to secondary bacterial challenge including limited window of type I IFN priming,^5^ anatomical locus of sensitization and challenge,^59^ critical cell types for type I IFN sensitization, and variable mechanisms that limit the duration and the magnitude of the inflammatory response.^73^

## 4 CONCLUSIONS

Overall, these data suggest that sufficiency of type I IFN priming in augmentation of inflammatory vigor, at least in conditions utilized in this report, is limited to certain secondary challenge.^5^ Further, our data provide novel insights into the mechanisms regulating the ability of different TLR2 ligands to augment inflammatory vigor and increase the risk of PTB.

Such findings are in agreement with the published reports, suggesting that diacylated lipopeptides are commonly present in Gram‐positive bacterial species correlated with PTB.^55^, ^56^, ^57^ Here, we demonstrate for the first time that: (1) activation of TLR2 by the synthetic lipopeptide Pam2Cys, but not Pam3Cys, induced PTB; (2) Pam2Cys challenge, compared to Pam3Cys, enhanced expression of IFN‐β and type I IFN signature genes; (3) IFNAR signaling is required for Pam2Cys‐ and Pam3Cys‐mediated proinflammatory cytokine production; (4) IFNAR signaling contributes to Pam2Cys‐driven induction of PTB; and (5) ability of IFN‐β priming/sensing is limited to certain molecular patterns as it was not sufficient to augment Pam3Cys‐driven inflammatory vigor and induction of PTB.

Our findings invoke several salient questions that remain to be elucidated including: Are specific Gram‐positive bacteria more likely to induce PTB because of expression of certain lipopeptides? Can certain lipopeptides induce protective effects by activating mechanisms that regulate overt inflammatory responses? Does TLR2 homodimerization or heterodimerization of TLR2 with TLR1 and TLR6 regulate the potency of immune response and induction of PTB? What are the critical TLR2 downstream pathway(s) that regulates the magnitude of inflammation? Do subclinical viral infections predispose to a secondary Gram‐positive challenge and thus increase the risk of augmented systemic inflammation and PTB? What is the contribution of fetal immune response to Gram‐positive bacteria? In summary, our novel data shed light on the complex nature of innate immune activation strength and argue that ability of pathogens to induce PTB, in an experimental setting, relies on the magnitude and type of TLR‐driven inflammation.
